# Seizures Following Carotid Endarterectomy: A Comprehensive Meta-Analysis of 69,479 Patients and Evidence-Based Recommendations for Perioperative Care

**DOI:** 10.3390/diagnostics15010006

**Published:** 2024-12-24

**Authors:** Kruthajn Rajesh, Helen Shen, Sonu M. M. Bhaskar

**Affiliations:** 1 Global Health Neurology Lab, Sydney, NSW 2150, Australia; 2 UNSW Medicine and Health, University of New South Wales (UNSW), South West Sydney Clinical Campuses, Sydney, NSW 2170, Australia; 3 Ingham Institute for Applied Medical Research, Clinical Sciences Stream, Sydney, NSW 2170, Australia; 4 NSW Brain Clot Bank, NSW Health Pathology, Sydney, NSW 2170, Australia; 5 Department of Neurology and Neurophysiology, Liverpool Hospital and South Western Sydney Local Health District (SWSLHD), Sydney, NSW 2170, Australia; 6 National Cerebral and Cardiovascular Center (NCVC), Department of Neurology, Division of Cerebrovascular Medicine and Neurology, Suita 564-8565, Osaka, Japan

**Keywords:** carotid endarterectomy, CEA, seizures, epilepsy, cerebrovascular disorders, cerebral hyperperfusion syndrome, stroke prevention

## Abstract

**Background:** Seizures are a rare but potentially serious complication following carotid endarterectomy (CEA). Understanding their prevalence and associated factors is crucial for optimizing perioperative care and improving patient outcomes. This meta-analysis aimed to estimate the pooled prevalence of seizures following CEA and explore clinical and procedural factors contributing to their occurrence. **Methods**: We conducted a systematic review and meta-analysis of studies reporting on seizures following CEA. A systematic search of PubMed, Embase, and Cochrane CENTRAL databases was performed, following PRISMA and MOOSE guidelines. Random-effects meta-analysis was used to calculate the pooled prevalence of postoperative seizures. Heterogeneity was assessed using the I^2^ statistic. A total of 20 studies, encompassing 69,479 patients, were included. **Results**: The overall pooled prevalence of seizures following CEA was 1% (95% CI: 0–2%; *p* < 0.001), with significant heterogeneity (I^2^ = 93.52%). Prospective studies reported a higher pooled prevalence (2%, 95% CI 0–4%; I^2^ = 76.34%) compared to retrospective studies (0%, 95% CI 0–1%; I^2^ = 91.51%). Male predominance was noted among patients who experienced seizures, and hypertension was the most common comorbidity. Cerebral hyperperfusion syndrome was identified as a key contributing factor to postoperative seizures. Data on long-term outcomes, including the development of epilepsy, were insufficient for further analysis. The methodological quality of the included studies varied, with most studies demonstrating a moderate risk of bias. **Conclusions**: Seizures occur in approximately 1% of patients following CEA, with higher rates observed in prospective studies. Cerebral hyperperfusion syndrome is an important contributor to this rare complication. We provide evidence-based specific recommendations for seizure management and introduce the SMART-CEA Checklist, a practical framework to guide perioperative care and reduce complications. Future research should focus on long-term outcomes, including epilepsy, and incorporate standardized methodologies to improve data reliability and guide clinical practice.

## 1. Background

Carotid endarterectomy (CEA) is a widely performed surgical procedure to mitigate the risk of ischemic stroke in patients with significant carotid artery stenosis [1]. By removing atherosclerotic plaques from the carotid artery, CEA improves blood flow and reduces cerebrovascular event recurrence [1]. Despite its proven efficacy in stroke prevention, the procedure is not without risks, with postoperative complications posing significant challenges to patient recovery [2]. Among these, seizures represent a rare but serious neurological complication with the potential to adversely impact patient outcomes and quality of life [3,4,5,6].

Seizures following CEA are believed to arise from cerebral hyperperfusion syndrome, a condition characterized by impaired cerebral autoregulation and excessive blood flow to previously hypoperfused brain regions [7]. This abrupt alteration in cerebral hemodynamics can result in neuronal hyperactivity, leading to seizures, cerebral edema, or, in severe cases, intracranial hemorrhage [8]. Hypertensive encephalopathy, another frequently implicated factor, may exacerbate the risk of seizures in the postoperative period. However, the exact mechanisms and risk factors underlying these events remain poorly understood [6]. While individual studies have reported on seizure occurrence post-CEA, no comprehensive synthesis of the available evidence has been conducted to elucidate the prevalence, clinical characteristics, and potential long-term consequences, such as the progression to epilepsy [4,5,9,10,11].

Understanding the prevalence and clinical burden of seizures following CEA is crucial for improving perioperative management strategies and informing clinical guidelines [12]. Seizures, even when transient, are associated with increased morbidity, longer hospital stays, and higher healthcare costs [13]. Identifying patients at higher risk for postoperative seizures could enable targeted interventions, including closer hemodynamic monitoring and tailored blood pressure management [14].

This study aims to address the existing knowledge gap by conducting a meta-analysis to estimate the pooled prevalence of seizures in patients undergoing CEA [8]. Additionally, we aim to explore the clinical characteristics of seizures, risk factors, and the potential progression to epilepsy. By synthesizing the available evidence, we seek to provide evidence-based insights that may guide therapeutic strategies, improve perioperative care, and inform future research in this critical area of interventional neuroradiology and vascular surgery.

### Objectives

This study aims to conduct a comprehensive meta-analysis to:

(1) Investigate the pooled prevalence of seizures following CEA.
(2) Analyze the potential progression of postoperative seizures to epilepsy.

## 2. Materials and Methods

### 2.1. Literature Search and Study Selection

**Figure 1:**
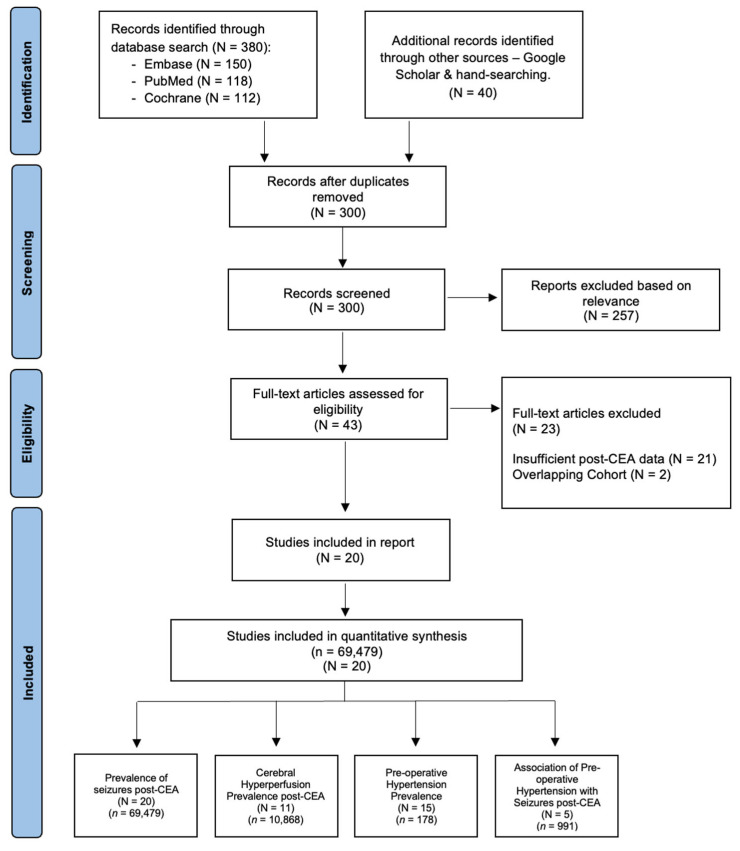
PRISMA flowchart of included studies in the meta-analysis of seizures following carotid endarterectomy (CEA). This figure illustrates the study selection process using the Preferred Reporting Items for Systematic Reviews and Meta-Analyses (PRISMA) framework, offering a clear depiction of the studies incorporated in the meta-analysis. Abbreviations: N—number of studies; *n*—cohort size; CEA—carotid endarterectomy.

A systematic search for relevant studies from 1 January 1980 to December 2024 was conducted using PubMed, Embase, and Cochrane Central Register of Controlled Trials (CENTRAL) databases. The search terms included “carotid endarterectomy”, “CEA”, “carotid surgery”, “endarterectomy”, “postoperative seizures”, “seizure”, “epilepsy”, “status epilepticus”, “hyperperfusion”, “cerebral hyperperfusion”, “postoperative hyperperfusion”, “cerebral reperfusion injury”, “hypertension”, “blood pressure”, “BP control”, “cerebral hypoperfusion”, “impaired vasoreactivity”, and “cerebral autoregulation”. A comprehensive and structured search strategy was implemented, with additional details provided in the Online Supplemental Information (Search Strategy). Studies not written in English or those not involving human participants were excluded. To identify further relevant studies, a manual review of references from key articles, systematic reviews, and meta-analyses was performed. The organization of studies and various subgroup analyses included in the meta-analysis is illustrated in a flowchart, following the Preferred Reporting Items for Systematic Reviews and Meta-Analyses (PRISMA) guidelines (Figure 1) [15]. Compliance with the 2020 checklist (Supplemental Table S1) [15] and the Meta-analysis of Observational Studies in Epidemiology (MOOSE) checklist (Supplemental Table S2) was ensured, with these documents available in the Online Supplemental Information. This study was registered in Open Science, registration number “hckr3” (https://osf.io/hckr3/ (accessed on 21 June 2024)).

### 2.2. Inclusion and Exclusion Criteria

Studies were eligible for inclusion if they met the following criteria: (a) patients diagnosed with seizures or with a history of epilepsy; (b) patients eligible for CEA; (c) participants aged 18 years or older; (d) reporting on post-CEA seizures or epilepsy or cerebral hyperperfusion syndrome or related complications (e.g., hypertension, hypoperfusion); reported data on pre-operative or postoperative blood pressure management; (e) employed a robust methodological design, including prospective or retrospective observational studies, randomized controlled trials, or meta-analyses; and (f) included a minimum sample size of 20 patients per group to ensure statistical reliability. Studies were excluded if they met any of the following criteria: (a) non-human or preclinical studies; (b) studies with overlapping datasets or duplicate publications; (c) studies where the full-text article was unavailable; (d) studies presented solely in abstract form without sufficient data on CEA-related outcomes or postoperative complications; (e) studies with anecdotal evidence, case reports, or editorials lacking robust data for meta-analysis; and (f) studies with unclear or inconsistent definitions of seizures or epilepsy. Seizures were defined as excessive hypersynchronous neuronal discharge in the brain, leading to a paroxysmal alteration of neurologic function, consistent with established clinical criteria to ensure uniformity across included studies [16]. Hypertension was defined according to the American Heart Association (AHA) guidelines as a blood pressure of ≥130/80 mmHg, providing a standardized threshold for identifying hypertensive patients [17]. Postoperative cerebral hyperperfusion syndrome was characterized as a greater-than-100% increase in cerebral blood flow or middle cerebral artery velocity compared to pre-operative baseline levels, as measured using transcranial Doppler or perfusion imaging [18].

### 2.3. Data Extraction and Methodological Quality Assessment

A meticulous data extraction and methodological quality assessment process was conducted to ensure the reliability and validity of the findings. EndNote v. 21.0 software (Clarivate Analytics, London, UK) was used to manage references and screen titles and abstracts. Articles that did not meet the eligibility criteria were excluded during this initial screening phase. Two authors independently performed the screening, and any discrepancies were resolved through discussion or consultation with a third reviewer. Articles that passed the initial screening were further assessed for inclusion in the systematic review or meta-analysis based on pre-defined eligibility criteria.

A standardized data extraction form was used to systematically collect key information from each included study. The extracted data included: (a) Study characteristics: author, year of publication, country, and study design (prospective or retrospective). (b) Patient demographics: age, gender distribution, sample size, and clinical characteristics of patients’ CEA. (c) CEA details: procedural characteristics, perioperative management protocols, and follow-up durations. (d) Seizure outcomes: prevalence of seizures, seizure subtypes (e.g., focal, generalized), severity (e.g., status epilepticus), and progression to epilepsy, if reported. (e) Risk factors and complications: data on cerebral hyperperfusion syndrome, hypertension, and blood pressure control.

The methodological quality of the included studies was assessed using the modified Jadad scale (MJA), which evaluates study design, randomization, blinding, and reporting of withdrawals or dropouts. Each study was assigned a quality score, with higher scores indicating better methodological rigor. The results of the quality assessment are summarized in Supplemental Table S3. Two independent reviewers conducted the quality assessment, and disagreements were resolved through joint discussions to reach consensus.

To address the study objectives, data were collected on the total number of patients undergoing CEA, the proportion who experienced postoperative seizures (prevalence), and those who developed epilepsy over time (incidence). However, during data extraction, it became evident that most studies lacked sufficient longitudinal data to reliably assess the progression from seizures to epilepsy. As a result, the analysis primarily focused on the prevalence of seizures and associated risk factors following CEA. The incidence of epilepsy was identified as an area for future investigation. The data extraction process adhered to the PRISMA guidelines, and compliance with the PRISMA 2020 checklist and the MOOSE guidelines was ensured.

### 2.4. Statistical Methodology

Statistical analyses were conducted using STATA v. 13.0 (StataCorp, College Station, TX, USA). Baseline data were extracted from all included studies, and descriptive statistics were used to summarize patient demographics, study characteristics, and seizure prevalence. For studies reporting medians and interquartile ranges (IQRs), means and standard deviations (SDs) were estimated using the method proposed by Wan et al. [19]. Adjustments to 95% confidence intervals (95% CIs) were made using the ‘cimethod (exact)’ and ’ftt’ commands in STATA. A random-effects model was employed to pool prevalence estimates of seizures following CEA, accounting for between-study variability. The random-effects model was chosen due to the anticipated heterogeneity across studies in terms of study design, patient populations, and diagnostic criteria for seizures. The pooled prevalence was reported as a percentage with 95% CIs.

Four types of subgroup analyses were conducted to explore the prevalence and associations related to seizures following CEA: (1) the pooled prevalence of seizures post-CEA; (2) the prevalence of pre-operative hypertension among patients who experienced seizures post-CEA; (3) the prevalence of cerebral hyperperfusion post-CEA; and (4) the association between pre-operative hypertension and seizures post-CEA. The organization of these subgroup analyses and the study selection process is illustrated in Figure 1. Heterogeneity across studies was assessed using the I^2^ statistic, which quantifies the proportion of total variation in effect estimates due to between-study heterogeneity rather than chance. Heterogeneity was categorized as follows: low: I^2^ < 40%; moderate: I^2^ = 30–60%; substantial: I^2^ = 50–90%; considerable: I^2^ = 75–100%. To explore potential sources of heterogeneity, subgroup analyses and sensitivity analyses were performed.

Subgroup analyses were conducted to investigate differences in seizure prevalence based on the following: *study design*: prospective vs. retrospective studies; *follow-up duration*: short-term (≤7 days) vs. longer-term (>7 days) follow-up; and *geographic region*: studies conducted in different regions to account for variations in clinical practice and patient populations.

Sensitivity analyses were performed by excluding studies with high risk of bias (as determined by the modified Jadad scale) to assess the robustness of the pooled prevalence estimate. Additionally, a leave-one-out analysis was conducted to evaluate the influence of individual studies on the overall results.

Publication bias was assessed using funnel plots and Egger’s test for small-study effects. Asymmetry in the funnel plot would suggest potential publication bias, which was further quantified using Egger’s regression test. A *p*-value < 0.05 was considered indicative of significant publication bias.

All statistical tests were two-tailed, and a *p*-value < 0.05 was considered statistically significant. Confidence intervals were reported at the 95% level.

## 3. Results

### 3.1. Description of Included Studies

A total of 20 studies [4,5,6,8,9,10,11,20,21,22,23,24,25,26,27,28,29,30,31,32], encompassing 69,479 patients, were included in this meta-analysis. The cohort sizes varied widely, ranging from 25 to 51,001 patients. The incidence of seizures post-CEA was low, with reported rates ranging from 0.01% to 13%. The mean age of patients was 67.7 years, with SDs reported between 8.7 and 15 years across studies. Male predominance was noted in most studies where gender data were available, particularly among patients who experienced seizures. Hypertension was the most common comorbidity, affecting 71.9% of patients, followed by coronary artery disease (23%). The prevalence of previous stroke or transient ischemic attack (TIA) varied significantly, with some studies reporting rates as high as 70%. Smoking was prevalent in 8 cohorts [8,9,11,22,23,28,31,32], while diabetes was reported in 10 cohorts [4,5,8,9,11,22,23,28,31,32].

Follow-up durations for seizure detection post-CEA ranged from 1 to 8 days, reflecting variability in study designs and monitoring protocols. Detailed clinical characteristics of the included studies are presented in Table 1. The methodological quality of the studies, assessed using the modified Jadad scale, revealed variable quality and risk of bias, as summarized in Supplemental Table S3. Funding bias was identified in only one study (Buczek et al. [5]). These findings highlight the heterogeneity in study designs, patient populations, and reporting practices, underscoring the need for standardized methodologies in future research.

### 3.2. Overall Prevalence of Seizures in Patients Undergoing CEA

**Figure 2:**
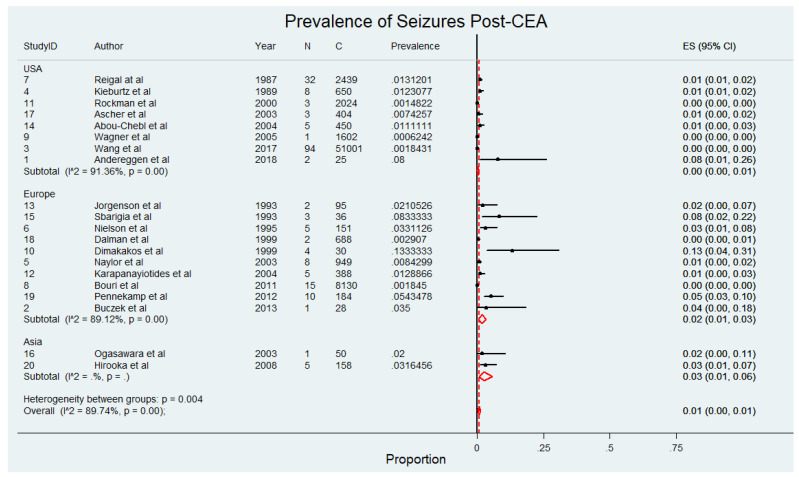
Pooled prevalence of seizures following carotid endarterectomy (CEA) across included studies [4,5,6,8,9,10,11,20,21,22,23,24,25,26,27,28,29,30,31,32]. This figure displays the prevalence of seizures in patients after receiving carotid endarterectomy. Abbreviations: CEA—carotid endarterectomy; N—number of patients with seizures; C—overall cohort; ES—effect size; CI—confidence interval; I^2^—the proportion of total variation in effect estimate due to between-study heterogeneit.

The meta-analysis presented in Figure 2, comprising 69,479 patients within 20 studies [4,5,6,8,9,10,11,20,21,22,23,24,25,26,27,28,29,30,31,32], evaluates the prevalence of seizures in patients undergoing CEA. The findings indicate that the overall prevalence of seizures post-CEA is low, with prospective studies reporting slightly higher rates compared to retrospective studies. Specifically, seizure prevalence in prospective studies ranged from 0% to 13%, with a pooled prevalence of 2% (95% CI 0–4) and considerable heterogeneity (I^2^ = 76.34). Retrospective studies reported seizure rates between 0% and 1%, with a pooled prevalence of 0% (95% CI 0–1) and considerable heterogeneity (I^2^ = 91.51). When data from all included studies were combined, the overall pooled prevalence of seizures post-CEA was 1% (95% CI 0–2; *p* < 0.001), with significant heterogeneity (I^2^ = 93.52). The pooled prevalence of seizures following CEA, stratified by study design, revealed slightly higher rates in prospective studies compared to retrospective studies. This stratified analysis is presented in Supplemental Figure S3.

**Table 1:** Characteristics of included studies in the meta-analysis of seizures following carotid endarterectomy.

Study ID	Author	Year	Region	Study Type	Sample Size (*n*)	Seizure Prevalence (*n*, %)	Follow-Up Duration (Days)	Age (Years ± SD)	Male (*n*, %) *	AF (*n*, %)	HL (*n*, %)	HTN (n, %)	CAD (*n*, %)	Previous stroke/TIA (*n*, %)	Smoking (*n*, %)	Diabetes (*n*, %)
1	Andereggen et al. [11]	2018	USA	Prospective	25	2 (8.0)	1–5	71.0 ± 8.7	18 (72.0)	-	20 (80.0)	20 (80.0)	9 (36.0)	17 (68.0)	17 (68.0)	8 (32.0)
2	Buczek et al. [5]	2013	Europe	Prospective	28	1 (3.6)	2–5	70.2 ± 9.4	23 (82.1)	-	-	22 (78.6)	-	11 (39.3)	-	8 (28.6)
3	Wang et al. [9]	2017	USA	Retrospective	51,001	94 (0.2)	3–6	70.2 ± 9.4	30,804 (60.4)	-	-	45,238 (88.7)	14,586 (28.6)	15,096 (29.6)	24,276 (47.6)	17,697 (34.7)
4	Kieburtz et al. [8]	1989	USA	Retrospective	650	8 (1.2)	7	69.5 ± 10.0	-	-	-	488 (75.1)	-	81 (12.5)	569 (87.5)	81 (12.5)
5	Naylor et al. [6]	2003	Europe	Prospective	949	8 (0.8)	2–8	65.8 ± 10.4	4 (0.4) **^œ^**	-	-	-	-	-	-	-
6	Nielson et al. [4]	1995	Europe	Prospective	151	5 (3.3)	5–7	62.0	97 (64.2)	-	-	-	40 (26.0)	59 (39.0)	-	17 (11.0)
7	Reigal et al. [10]	1987	USA	Retrospective	2439	32 (1.3)	1–7	-	-	-	-	-	-	-	-	-
8	Bouri et al. [30]	2011	Europe	Retrospective	8130	15 (0.2)	3–6	66.0 ± 9.5	-	-	-	1545 (19.0)	-	-	-	-
9	Wagner et al. [20]	2005	USA	Retrospective	1602	1 (0.1)	1–7		-	-	-	-	-	224 (14.0)		
10	Dimakakos et al. [28]	1999	Europe	Prospective	30	4 (13.3)	1–7	66.6 ± 15.0	22 (73.0)	-	18 (60.0)	17 (57.0)	17 (57.0)		15 (50.0)	5 (17.0)
11	Rockman et al. [22]	2000	USA	Retrospective	2024	3 (0.1)	1–7	68.7 ± 9.4	1295 (64.0)	-	-	1416 (70.0)	951 (47.0)	850 (42.0)	971 (48.0)	506 (25.0)
12	Karapanayiotides et al. [25]	2004	Europe	Prospective	388	5 (1.3)	2–7	70.4 ± 6.2	77 (20.0)	-	-	230 (59.0)	-			
13	Jorgenson et al. [26]	1993	Europe	Prospective	95	2 (2.1)	1–14	59.0 ±12.0	62 (65.0)	-	-	44 (46.0)	-	48 (51.0)		
14	Abou-Chebl et al. [32]	2004	USA	Retrospective	450	5 (1.1)	1–4	72.7 ± 10.9	-	-	197 (44.0)	339 (75.0)	255 (57.0)		225 (50.0)	176 (39.0)
15	Sbarigia et al. [21]	1993	Europe	Prospective	36	3 (8.3)	1–2	67.0 ± 6.0	32 (89.0)	-	-	27 (75.0)	-			
16	Ogasawara et al. [24]	2003	Asia	Prospective	50	1 (2.0)	1–6	68.6 ± 5.8	44 (88.0)	-	-	-	-			
17	Ascher et al. [31]	2003	USA	Prospective	404	3 (0.7)	1–8	69.0 ± 8.0	221 (55.0)	-	-	275 (68.0)	98 (24.0)	129 (32.0)	176 (44.0)	122 (30.0)
18	Dalman et al. [29]	1999	Europe	Prospective	688	2 (0.3)	1–7	69.0 ± 10.3	447 (65.0)	-	-	-	-			
19	Pennekamp et al. [23]	2012	Europe	Prospective	184	10 (5.4)	1–7	68.8 ± 10.9	141 (77.0)	-	164 (89.0)	135 (73.0)	50 (27.0)		61 (33.0)	36 (20.0)
20	Hirooka et al. [27]	2008	Asia	Prospective	158	5 (3.2)	1–7	67.2 ± 6.5	150 (95.0)	-	-	128 (81.0)	-	111 (70.0)	-	-

Abbreviations: *n*—number of patients; SD—standard deviation; AF—atrial fibrillation; HL—hyperlipidaemia; HTN—hypertension; CAD—coronary artery disease; TIA—transient ischemic attack. * Note: Demographics are based on the entire cohort. Clinical demographics (e.g., ‘Male’) are based on the subset of patients who experienced a seizure post-CEA).

### 3.3. Prevalence of Pre-Operative Hypertension Among Patients Who Experienced Seizures Post-CEA

**Figure 3:**
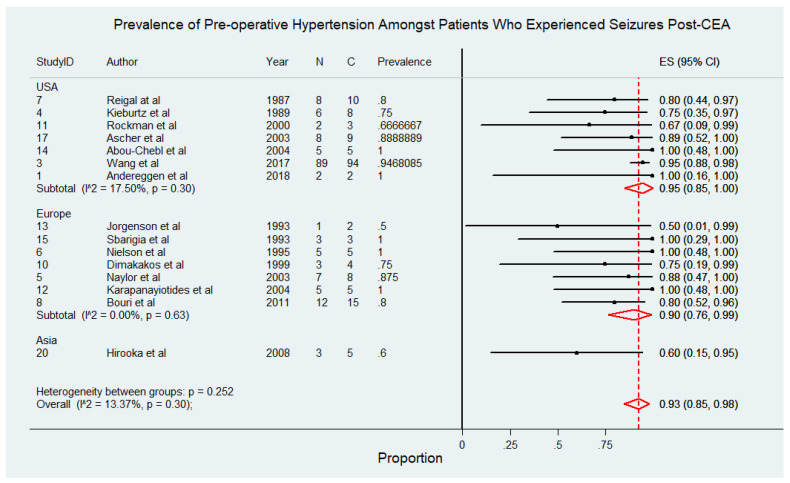
Prevalence of pre-operative hypertension among patients who experienced seizures following carotid endarterectomy (CEA) [4,6,8,9,10,11,21,22,25,26,27,28,30,31,32]. Abbreviations: CEA—carotid endarterectomy; N—number of patients with seizures; C—overall cohort; ES—effect size; CI—confidence interval; I^2^—the proportion of total variation in effect estimate due to between-study heterogeneity.

This subgroup comprised 178 patients drawn from 15 studies [4,6,8,9,10,11,21,22,25,26,27,28,30,31,32]. Figure 3 depicts the prevalence of pre-operative hypertension among patients who experienced seizures following CEA. The findings reveal that pre-operative hypertension is a highly prevalent comorbidity in this patient population, underscoring its potential role as a significant risk factor for postoperative seizures. In studies conducted in the USA, the prevalence of pre-operative hypertension ranged from 67% to 100%, with a pooled prevalence of 95% (95% CI: 85% to 100%) and low heterogeneity (I^2^ = 17.5%). Similarly, European studies reported prevalence rates between 50% and 100%, with a pooled prevalence of 90% (95% CI: 76% to 99%) and no observed heterogeneity (I^2^ = 0%). In contrast, studies conducted in Asia reported a lower prevalence of 60% (95% CI: 15% to 95%), although this estimate was based on a smaller number of studies, limiting its generalizability. The overall pooled prevalence of pre-operative hypertension among patients with post-CEA seizures was 93% (95% CI: 85% to 98%), with low heterogeneity (I^2^ = 13.37%), indicating consistency across most included studies. The pooled prevalence of pre-operative hypertension among patients who experienced seizures following CEA, stratified by study design, demonstrated consistent findings across both prospective [91%, 95% CI 77–100%; I^2^ = 0%] and retrospective [89%, 95% CI 76–99%; I^2^ = 43.95%] studies. These results are detailed in Supplemental Figure S4.

### 3.4. Prevalence of Cerebral Hyperperfusion Post-CEA

**Figure 4:**
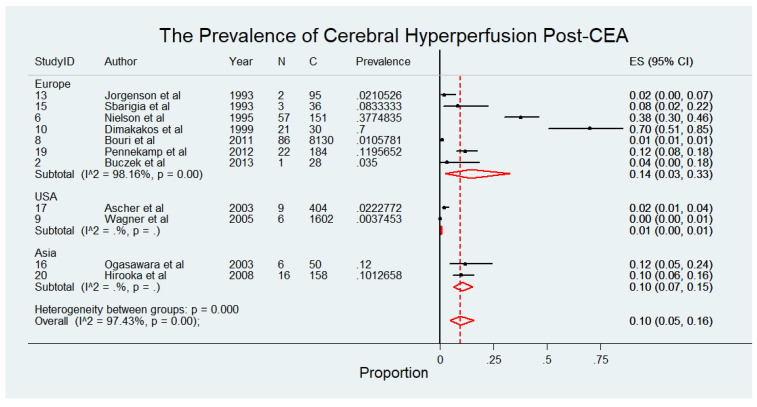
Prevalence of cerebral hyperperfusion syndrome following carotid endarterectomy (CEA) [4,5,20,21,23,24,26,27,28,30,31]. Abbreviations: CEA—carotid endarterectomy; N—number of patients with seizures; C—overall cohort; ES—effect size; CI—confidence interval; I^2^—the proportion of total variation in effect estimate due to between-study heterogeneity.

The meta-analysis presented in Figure 4 comprises 10,868 patients from 11 studies [4,5,20,21,23,24,26,27,28,30,31], evaluating the prevalence of cerebral hyperperfusion among patients following CEA. The findings reveal notable regional variations in the reported prevalence of cerebral hyperperfusion, with higher rates observed in European studies compared to those from other regions. Specifically, European studies reported prevalence rates ranging from 2% to 70%, with a pooled prevalence of 14% (95% CI: 3% to 33%) and considerable heterogeneity (I^2^ = 98.16%), reflecting variability in study populations and methodologies. In Asia, the pooled prevalence was 10% (95% CI: 7% to 15%) with no observed heterogeneity, suggesting more consistent findings across studies in this region. Conversely, studies conducted in the USA reported significantly lower prevalence rates, with a pooled prevalence of 1% (95% CI: 0% to 1%) with no heterogeneity reported. Overall, the pooled prevalence of cerebral hyperperfusion post-CEA across all studies was 10% (95% CI: 5% to 16%), with considerable heterogeneity (I^2^ = 97.43%). The pooled prevalence of cerebral hyperperfusion syndrome following CEA, stratified by study design, highlighted significant variability between prospective [14%, 95% CI 5–26%; I^2^ = 95.6%] and retrospective [1%, 95% CI 1–1%; I^2^ = 0%] studies. This analysis is illustrated in Supplemental Figure S5.

### 3.5. Association Between Pre-Operative Hypertension and Seizures Post-CEA

**Figure 5:**
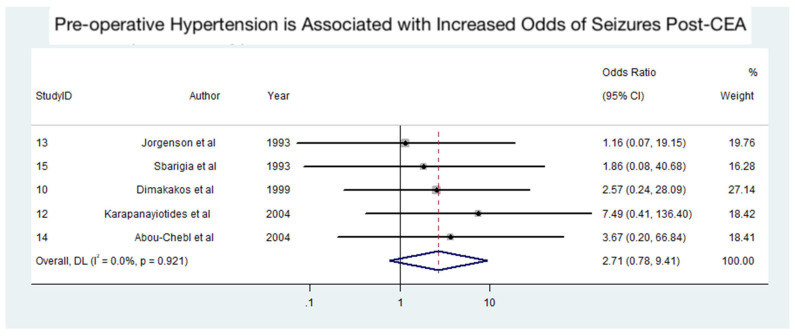
Association between pre-operative hypertension and seizures following carotid endarterectomy (CEA) [21,25,26,28,32]. Abbreviations: CEA—carotid endarterectomy; CI—confidence interval; DL—DerSimonian and Laird method; I^2^—the proportion of total variation in effect estimate due to between-study heterogeneity.

Figure 5, consisting of 19 patients from five studies [21,25,26,28,32], portrays the findings of the meta-analysis on the association between pre-operative hypertension and the odds of developing seizures after CEA. The analysis suggests that pre-operative hypertension may be associated with an increased risk of seizures post-CEA, although the association was not statistically significant. The pooled odds ratio (OR) was 2.71 (95% CI: 0.78–9.41, *p* > 0.05), indicating a potential trend toward increased risk. However, the wide confidence intervals reflect variability in sample sizes and effect estimates across the included studies. Importantly, no heterogeneity was observed among the studies (I^2^ = 0%, *p* = 0.921), suggesting consistency in the reported findings despite the lack of statistical significance. The influence of a single study on the meta-analysis for the association between pre-operative hypertension and seizures post-CEA is illustrated in Supplemental Figure S1. The funnel plot with pseudo 95% confidence limits for the meta-analysis is presented in Supplemental Figure S2.

## 4. Discussion

Our study is the first to provide evidence-based insights into the prevalence of seizures following CEA, identifying this rare but clinically significant complication in approximately 1% of patients. These findings underscore the necessity of personalized treatment strategies for a small albeit high-risk subgroup. Pre-operative hypertension, with a pooled prevalence of 93% among patients who experienced seizures, emerged as a key predisposing factor, further associated with elevated odds of postoperative seizures. This association suggests a potential mechanistic link between hypertension-related cerebral dysregulation and adverse perioperative neurological outcomes.

Cerebral hyperperfusion syndrome (CHS), identified in 10% of patients, displayed substantial geographic variability, likely reflecting differences in diagnostic definitions and criteria across studies. The high heterogeneity in European studies particularly underscores the need for standardized diagnostic approaches and consistent monitoring protocols. These findings collectively emphasize the critical importance of stringent perioperative blood pressure control and the implementation of tailored risk mitigation strategies in vulnerable patients.

The observed differences in seizure prevalence between study designs may reflect variations in monitoring intensity, follow-up durations, and diagnostic rigor. Prospective studies, with standardized protocols, reported a broader range of seizure events compared to retrospective analyses, which may be limited by incomplete or underreported data. Despite the low overall prevalence, the significant heterogeneity observed calls for further investigation into the interplay between CHS, perioperative hypertension, and patient-specific risk factors.

The geographic differences in CHS prevalence likely stem from variations in surgical techniques, patient selection, and perioperative management protocols. Tailored approaches—including strict perioperative blood pressure control, routine use of transcranial Doppler, and advanced imaging—may reduce CHS incidence and its associated complications, including seizures and intracranial hemorrhage. While the evidence supporting pre-operative hypertension as a predictor of postoperative seizures remains inconclusive, its high prevalence in this cohort underscores the need for aggressive yet cautious management. Gradual management may be more appropriate in cases of severe symptomatic carotid stenosis to mitigate the risk of stroke progression [33]. These findings reinforce the critical need for optimizing blood pressure management in the perioperative period to improve outcomes and minimize complications. Standardized reporting practices and extended follow-up protocols are essential for future research to refine our understanding of this rare complication. This will enhance our ability to stratify risk, develop targeted interventions, and ensure optimal perioperative care for patients undergoing CEA.

The pathophysiology of seizures following CEA is multifactorial and complex. Cerebral hyperperfusion syndrome has been identified as a primary contributor, leading to complications such as cerebral edema and dysregulation of the brain’s autoregulatory capacity [7,34]. This syndrome results from an abrupt increase in cerebral blood flow that the brain’s vascular system fails to modulate, causing increased intracranial pressure and subsequent neuronal damage [33]. Hypertension, particularly hypertensive encephalopathy, may also play a role [6], as it is characterized by a rapid onset of neurological symptoms, including seizures, due to severely elevated blood pressure. Elevated middle cerebral artery velocities (MCAVs) further complicate the clinical picture, blurring the lines between cause and effect. Both hyperperfusion and hypertension must be carefully managed post-CEA to reduce the risk of seizures and other complications [6,35].

Our study presents evidence-based insights into real-world prevalence data compared to previous reports, with an estimated pooled prevalence of 1% (range: 0–2%). While prior studies have broadly addressed cognitive decline and hyperperfusion syndrome, they have not specifically focused on seizure prevalence, highlighting a critical gap in the literature [36,37,38,39]. There is a substantial lack of data on the prevalence and management of epilepsy following CEA. Current AHA guidelines do not address protocols for managing patients at greater risk of postoperative seizures, underscoring the need for further research in this area [39].

Cerebral hyperperfusion syndrome, although rare, can lead to high mortality due to its association with intracranial hemorrhages. It typically presents as a unilateral headache, confusion, seizures, or focal neurological signs [40]. Treating physicians must remain vigilant for seizure development and implement appropriate management strategies.

Current management includes pre-operative assessments, peri-operative monitoring, and postoperative care. Pre-operative assessments should focus on identifying and managing uncontrolled hypertension, particularly in patients with systolic blood pressures above 180 mmHg [6]. Perioperative management involves maintaining normotension and normocarbia, adhering to anesthesia guidelines, and utilizing TCD monitoring to detect hyperperfusion [6]. Despite these measures, some patients may still develop seizures post-CEA, requiring prompt treatment with diazepam for seizures, labetalol for severe hypertension, and dexamethasone for cerebral edema [6]. CT imaging is essential to exclude intracranial hemorrhage in these cases [6,41]. Emerging evidence suggests that perioperative use of TCD monitoring can improve outcomes by identifying patients at risk of hyperperfusion and intracerebral hemorrhage [29].

In the broader context of carotid revascularization, alternative techniques such as carotid artery stenting (CAS) and transcarotid artery revascularization (TCAR) have emerged as viable options, particularly for high-risk patients [42]. While CAS is associated with a higher risk of stroke and embolization, it may have a lower incidence of cranial nerve injuries compared to CEA [43]. TCAR, a newer technique, combines aspects of both CEA and CAS, utilizing flow reversal to reduce embolic risk during stenting [44]. Early data suggest that TCAR may offer better outcomes in terms of stroke prevention, with fewer complications in high-risk patients [45].

However, data on seizure prevalence specific to CAS and TCAR remain sparse [46]. Given the role of hyperperfusion and embolic phenomena in seizure pathophysiology, these techniques may carry differential risks, warranting further investigation [47]. Future studies should systematically evaluate these differences to inform patient-specific treatment strategies.

### 4.1. Limitations

This study has several limitations. First, data on symptom severity, diagnostic duration, and seizure phenotype (e.g., epilepsy or status epilepticus) were insufficient across multiple studies, preventing a comprehensive meta-analysis. Discrepancies in sample sizes and demographic data across studies compromised the validity and accuracy of our pooled prevalence estimates. The lack of detailed information on patients’ past medical history of seizures, which could increase their risk, further limits the generalizability of our findings.

Considerable heterogeneity was observed among the included studies, suggesting they may have been estimating different quantities due to variations in study design, patient populations, and methodologies. The high heterogeneity observed in the meta-analyses, particularly for pooled prevalence estimates of seizures and cerebral hyperperfusion syndrome, suggests significant variability in study designs, patient populations, and diagnostic criteria, which may have impacted the reliability of the findings. Furthermore, the inclusion of studies spanning a wide time period introduces the possibility that advancements in surgical techniques, anesthesia protocols, and perioperative care processes may have influenced the reported prevalence of seizures. These temporal variations, along with individual patient differences, were not accounted for in the analysis, potentially impacting the consistency of the findings.

Another notable limitation is the lack of available data on pre-operative cerebral hypoperfusion or impaired vasoreactivity and their association with postoperative seizures following CEA. Despite their potential relevance, insufficient research has explored these variables comprehensively, leaving a significant gap in understanding their role as risk factors. Future studies should prioritize investigating cerebral hypoperfusion using advanced imaging modalities, such as MRI and CT perfusion, alongside other emerging biomarkers, to better identify patients at risk and develop targeted preventative strategies.

Systems-level factors, such as variations in treatment protocols, anesthesia techniques, and diagnostic procedures, also differed across studies. Previous evidence suggests that the type of anesthesia can impact clinical outcomes post-CEA [48], indicating the need for further research on the effects of anesthesia techniques on seizure risk [49]. Variations in the rigor of pre- and intra-operative assessments may also affect the reported prevalence of seizures. Additionally, the inconsistent reporting of long-term monitoring and follow-up durations for seizure detection post-CEA likely led to missed incidental seizures, further complicating the interpretation of results. The inconsistent reporting of key variables, such as follow-up durations, seizure detection methods, and regional differences in clinical practices, further complicates the interpretation and generalizability of the results. Since seizures are not commonly anticipated during CEA, the lack of consistent data collection and reporting practices may have introduced potential confounding effects.

### 4.2. Recommendations

Table 2 provides a comprehensive summary of specific recommendations for managing seizures and related complications following CEA, based on evidence and expert consensus. These recommendations address critical aspects of care, such as optimizing pre-operative blood pressure control (graded 1a for strong evidence) [30,38,50,51] and implementing smoking cessation programs pre-operatively (graded 2b for conditional evidence) [52,53,54,55]. The grading system highlights the strength and quality of evidence supporting each recommendation, ensuring clarity for clinical application.

To improve outcomes and address current gaps, future studies should focus on establishing standardized follow-up periods and implementing long-term monitoring protocols to capture delayed seizure events. Consistent reporting guidelines, including detailed documentation of follow-up timeframes and seizure occurrences, are essential for improving data reliability. Multi-center collaborations can help validate findings across diverse populations, while advanced monitoring techniques, such as TCD and quantitative MRI, may enhance risk stratification. Additionally, educating patients on self-monitoring and clearly defining seizure types across studies will improve comparability and guide clinical decision making. By addressing these limitations, future research can refine management strategies and optimize patient care following CEA.

### SMART-CEA Checklist: A Practical Framework for Preventing and Managing Postoperative Seizures Following Carotid Endarterectomy

**Figure 6:**
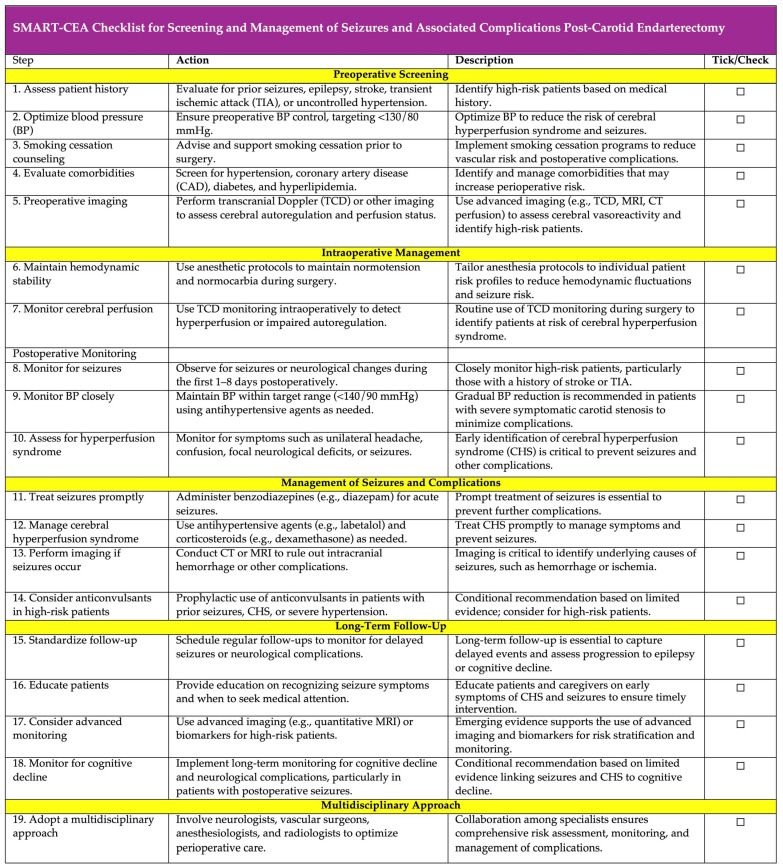
SMART-CEA Checklist: a practical guide for screening and management of seizures and associated complications post-carotid endarterectomy. SMART-CEA stands for: S—**Screening:** Assess risk factors such as hypertension, prior seizures, TIA, and smoking; M—**Monitoring:** Monitor cerebral perfusion and blood pressure intraoperatively and postoperatively; A—**Assessing:** Assess for hyperperfusion syndrome and seizure symptoms; R—**Responding:** Respond promptly to seizures and complications with appropriate interventions; T—**Tailoring:** Tailor long-term follow-up and patient education for seizure prevention and management; and CEA—carotid endarterectomy. The SMART-CEA checklist provides a practical, step-by-step guide for clinicians to ensure comprehensive screening, monitoring, and management of seizures and associated complications in patients undergoing CEA. Each step includes a tick/check option for easy tracking and implementation in clinical workflows. Abbreviations: AF—atrial fibrillation; HL—hyperlipidaemia; HTN—hypertension; CAD—coronary artery disease; TIA—transient ischaemic attack; CEA—carotid endarterectomy; TCD—transcranial Doppler; BP—blood pressure; CT—computed tomography; MRI—magnetic resonance imaging.

To bridge existing gaps and build upon evidence-based recommendations, we present the SMART-CEA Checklist (Figure 6; also available as Supplemental S2) as a practical framework for clinicians to optimize the screening and management of seizures and associated complications following CEA. This tool integrates key findings from our meta-analysis and the existing literature, focusing on pre-operative risk assessment, perioperative monitoring, and postoperative care.

The acronym “SMART” encapsulates five critical steps: screening for risk factors, monitoring cerebral perfusion and blood pressure, assessing for hyperperfusion syndrome and seizure symptoms, responding promptly to complications with appropriate interventions, and tailoring long-term follow-up and education. By offering clear, actionable steps, the checklist aims to standardize care processes, facilitate early detection of complications such as CHS, and ensure timely and effective management of seizures.

Designed to be user-friendly and easily incorporated into routine clinical workflows, the SMART-CEA Checklist has the potential to improve patient outcomes significantly. However, its implementation requires further validation across diverse clinical settings to assess its broader applicability and ensure its effectiveness in optimizing perioperative care.

## 5. Conclusions

In conclusion, seizures following CEA are rare but clinically significant, with an overall pooled prevalence of 1%, as demonstrated in this meta-analysis of 69,479 patients. The slightly higher prevalence observed in prospective studies and the identification of cerebral hyperperfusion syndrome as a key contributing factor emphasize the need for diligent perioperative blood pressure management and close neurological monitoring. To address these risks, we provide evidence-based specific recommendations and introduce the SMART-CEA Checklist, a practical framework designed to guide clinicians in optimizing perioperative care and reducing complications. However, significant heterogeneity across studies and limited data on long-term outcomes, such as progression to epilepsy, highlight the need for standardized diagnostic criteria, consistent follow-up protocols, and multi-center collaborations. Future research should focus on improving data reliability, identifying reliable predictors, and exploring long-term outcomes to enhance personalized care and optimize patient outcomes.

## Figures and Tables

**Table 2:** Evidence-based recommendations for the management of seizures and related complications following carotid endarterectomy (CEA).

Specific Recommendation	Explanation	Grading	Grading Justification
Optimize pre-operative blood pressure (BP) control	Pre-operative BP control should be optimized, particularly in patients with hypertension, to reduce the risk of cerebral hyperperfusion syndrome and seizures post-CEA.	1b	Strong recommendation supported by good-quality evidence from observational studies and clinical guidelines showing hypertension as a significant risk factor for postoperative complications [38,56].
Routine use of transcranial Doppler (TCD) monitoring	Routine use of TCD monitoring during and after CEA to identify patients at risk of cerebral hyperperfusion syndrome.	2a	Moderate-quality evidence from observational studies and clinical practice guidelines demonstrating the utility of TCD in detecting hyperperfusion and preventing complications [29,57,58].
Monitor patients with history of stroke or transient ischemic attack (TIA) postoperatively	Patients with a history of stroke or TIA should be closely monitored postoperatively for seizures, as they represent a high-risk subgroup.	2b	Moderate-quality evidence from observational studies indicates that prior cerebrovascular events increase the risk of seizures post-CEA, though further research is needed to confirm causality [59].
Implement smoking cessation programs pre-operatively	Smoking cessation programs should be implemented pre-operatively for patients undergoing CEA to reduce overall vascular risk and potential postoperative complications.	2b	Weaker recommendation based on lower-quality evidence from observational studies linking smoking to worse vascular outcomes, though direct evidence for seizure prevention is limited [52,53,54,55].
Standardize long-term follow-up for seizure development post-CEA	Long-term follow-up and monitoring for seizure development post-CEA should be standardized, extending beyond the immediate postoperative period to capture delayed events.	2c	Conditional recommendation based on emerging evidence and expert consensus, as current studies lack consistent long-term follow-up data to assess seizure progression or epilepsy development [60].
Use perioperative anesthetic protocols to maintain normotension and normocarbia	Use of perioperative anesthetic protocols that maintain normotension and normocarbia to minimize the risk of cerebral hyperperfusion syndrome and associated seizures.	2a	Moderate-quality evidence from observational studies and clinical practice guidelines regarding perioperative management studies showing that maintaining stable hemodynamics reduces the risk of hyperperfusion and seizures [61,62].
Treat cerebral hyperperfusion syndrome with antihypertensive agents and sedatives	Patients with cerebral hyperperfusion syndrome should be promptly treated with antihypertensive agents (e.g., labetalol) or sedatives (e.g., Dexmedetomidine) to manage symptoms and prevent seizures.	2b	Moderate-quality evidence from observational studies and clinical practice guidelines supports the use of these interventions, though randomized controlled trials are lacking [47,63].
Future studies should focus on identifying biomarkers or imaging predictors	Future studies should focus on identifying biomarkers or imaging predictors (e.g., quantitative MRI or TCD) for seizure risk stratification in patients undergoing CEA.	Not graded	No specific grade assigned due to the lack of direct evidence; this recommendation reflects the need for further research to improve risk prediction and patient outcomes [57,64,65].
Early identification and management of cerebral hyperperfusion syndrome (CHS)	Implement early identification protocols for CHS using TCD or advanced imaging modalities (e.g., MRI, CT perfusion) to detect hyperperfusion and prevent complications such as seizures.	2a	Moderate-quality evidence supports the use of TCD and imaging for identifying CHS, though further research is needed to standardize protocols and validate their effectiveness [29,58,66].
Gradual blood pressure reduction in severe symptomatic carotid stenosis	Gradually reduce blood pressure in patients with severe symptomatic carotid stenosis to minimize the risk of stroke progression and hyperperfusion syndrome.	2a	Observational studies and expert consensus suggest that gradual blood pressure management reduces complications, though high-quality RCTs are lacking [61].
Use of anticonvulsants in high-risk patients	Consider prophylactic use of anticonvulsants in high-risk patients (e.g., those with prior seizures, CHS, or severe hypertension) undergoing CEA.	2c	Conditional recommendation based on limited evidence and expert consensus, with a need for further research to establish efficacy and safety [61].
Tailored perioperative anesthesia protocols	Tailor anesthesia protocols to individual patient risk profiles, including the use of regional anesthesia where appropriate, to reduce hemodynamic fluctuations and seizure risk.	2b	Moderate-quality evidence supports the benefits of regional anesthesia, though its applicability may vary based on patient and procedural factors [6,48,49].
Patient education on postoperative symptoms	Educate patients and caregivers on recognizing early symptoms of CHS and seizures (e.g., severe headache, confusion, focal neurological deficits) to ensure prompt medical attention.	2c	Conditional recommendation based on expert consensus, as evidence on the impact of patient education on outcomes is limited [67,68].
Standardized use of advanced imaging for risk stratification	Incorporate advanced imaging techniques (e.g., quantitative MRI, CT perfusion) preoperatively to assess cerebral vasoreactivity and identify patients at high risk for postoperative seizures.	2c	Emerging evidence supports the utility of advanced imaging, but further studies are needed to validate its role in routine clinical practice [64,69].
Multidisciplinary approach to perioperative care	Adopt a multidisciplinary approach involving neurologists, vascular surgeons, anesthesiologists, and radiologists to optimize perioperative care and reduce seizure risk.	Not graded	No specific evidence available to grade this recommendation, but it reflects expert consensus and best practices in perioperative care.
Long-term monitoring for cognitive decline	Implement long-term monitoring for cognitive decline and neurological complications in patients undergoing CEA, particularly those with postoperative seizures.	2c	Conditional recommendation based on limited evidence linking seizures and CHS to cognitive decline, with a need for further research [70].

Recommendations are based on the findings of the meta-analysis, published evidence and expert consensus. Implement long-term monitoring for cognitive decline and neurological complications in patients undergoing CEA, particularly those with postoperative seizures. Grading reflects the strength of the recommendation and the quality of supporting evidence, as outlined in the grading system. Emerging evidence and further research may refine these recommendations. Explanation of Grading: 1a: Strong recommendation with robust evidence from multiple randomized controlled trials (RCTs) or systematic reviews; 1b: Strong recommendation with good-quality evidence but some variability or limitations in study design or population; 2a: Weaker recommendation based on moderate-quality evidence, acknowledging certain limitations in the data; 2b: Weaker recommendation with lower-quality evidence, often from observational studies or less rigorous trials; 2c: Conditional recommendation based on emerging evidence or expert consensus, reflecting the need for further research; Not graded: Recommendations based on inconclusive or emerging evidence, often highlighting areas for future investigation. Abbreviations: TIA—transient ischemic attack; CEA—carotid endarterectomy; CHS—cerebral hyperperfusion syndrome; TCD—transcranial Doppler; BP—blood pressure; MRI—magnetic resonance imaging.

## Data Availability

The original contributions presented in the study are included in the article and Online Supplemental Information, and further inquiries can be directed to the corresponding author.

## Supplementary Materials

The following supporting information can be downloaded at: https://www.mdpi.com/article/10.3390/diagnostics15010006/s1, Table S1: PRISMA checklist for the meta-analysis of seizures following carotid endarterectomy; Table S2: MOOSE checklist for meta-analyses of observational studies included in the study on seizures post-carotid endarterectomy; Table S3: Jaded analysis for methodological quality, risk of bias, and test for funding bias in the meta-analysis; Figure S1: Influence of a single study on the meta-analysis of the association between pre-operative hypertension and seizures following carotid endarterectomy; Figure S2: Funnel plot with pseudo 95% confidence limits for the meta-analysis of seizures post-carotid endarterectomy; Figure S3. Meta-analysis of pooled prevalence of seizures following carotid endarterectomy stratified by study design (prospective vs. retrospective); Figure S4. Meta-analysis of pooled prevalence of pre-operative hypertension among patients who experienced seizures following carotid endarterectomy stratified by study design (prospective vs. retrospective); Figure S5. Meta-analysis of pooled prevalence of cerebral hyperperfusion syndrome following carotid endarterectomy stratified by study design (prospective vs. retrospective); https://www.mdpi.com/article/10.3390/diagnostics15010006/s2, Supplemental SI2 SMART-CEA Checklist.
