# Supplementary material for: Seizures Following Carotid Endarterectomy: A Comprehensive Meta-Analysis of 69,479 Patients and Evidence-Based Recommendations for Perioperative Care
Source: Diagnostics (Basel). 2024 Dec 24;15(1):6. doi: 10.3390/diagnostics15010006 (PMC11840276; doi:10.3390/diagnostics15010006)

SUPPLEMENTAL INFORMATION

Seizures Following Carotid Endarterectomy: A Comprehensive Meta-Analysis of 69,479 Patients and Evidence-Based Recommendations for Perioperative Care

Authors: Kruthajn Rajesh, Helen Shen and Sonu M. M. Bhaskar*

*Correspondence to: Dr Sonu M. M. Bhaskar, MD PhD
Email: Sonu.Bhaskar@globalhealthneurolab.org / Bhaskar.Sonu@ncvc.go.jp

Table of Contents

2. Supplemental Tables 4

2.1. Supplemental Table S1. PRISMA checklist for the meta-analysis of seizures following carotid endarterectomy. 4

2.2. Supplemental Table S2. MOOSE checklist for meta-analyses of observational studies included in the study on seizures post-carotid endarterectomy 8

2.3. Supplemental Table S3. Jaded analysis for methodological quality, risk of bias, and test for funding bias in the meta-analysis. 10

3. Supplemental Figures 13

3.1. Supplemental Figure S1. Influence of a single study on the meta-analysis of the association between pre-operative hypertension and seizures following carotid endarterectomy. 13

3.2. Supplemental Figure S2. Funnel plot with pseudo 95% confidence limits for the meta-analysis of seizures post-carotid endarterectomy. 14

3.3. Supplemental Figure S3. Meta-analysis of pooled prevalence of seizures following carotid endarterectomy stratified by study design (prospective vs. retrospective). 15

3.4. Supplemental Figure S4. Meta-analysis of pooled prevalence of pre-operative hypertension among patients who experienced seizures following carotid endarterectomy stratified by study design (prospective vs. retrospective). 16

3.5. Supplemental Figure S5. Meta-analysis of pooled prevalence of cerebral hyperperfusion syndrome following carotid endarterectomy stratified by study design (prospective vs. retrospective). 17

1. Search strategy (Keywords/MeSH Terms)

PubMed:

((carotid endarterectomy[MeSH Terms] OR carotid endarterectomy OR CEA OR carotid surgery OR endarterectomy) AND
(seizure[MeSH Terms] OR seizures OR epilepsy OR postoperative seizures OR status epilepticus) AND
(cerebral hyperperfusion[MeSH Terms] OR hyperperfusion syndrome OR postoperative hyperperfusion OR cerebral reperfusion injury) AND
(hypertension[MeSH Terms] OR blood pressure OR labile blood pressure OR BP control)
AND (cerebral hypoperfusion OR impaired vasoreactivity OR cerebral autoregulation))

Filters applied: Clinical Study, Clinical Trial, Clinical Trial, Phase I, Clinical Trial, Phase II, Clinical Trial, Phase III, Clinical Trial, Phase IV, Comparative Study, Dataset, Evaluation Study, Meta-Analysis, Multicentre Study, Observational Study, Randomized Controlled Trial, Review, Systematic Review, Validation Study, Humans, English, Adult: 19+ years.

Embase:

('carotid endarterectomy'/exp OR 'CEA'/exp OR 'carotid surgery'/exp OR 'endarterectomy'/exp) AND ('postoperative seizures'/exp OR 'seizure'/exp OR 'epilepsy'/exp) AND ('cerebral hyperperfusion'/exp OR 'hyperperfusion syndrome'/exp OR 'postoperative hyperperfusion'/exp OR 'cerebral reperfusion injury'/exp) AND ('hypertension'/exp OR 'blood pressure'/exp OR 'labile blood pressure'/exp OR 'BP control'/exp) AND ('cerebral hypoperfusion'/exp OR 'impaired vasoreactivity'/exp OR 'cerebral autoregulation'/exp)

Cochrane:

(carotid endarterectomy OR CEA OR carotid surgery OR endarterectomy) AND (postoperative seizures OR seizure OR epilepsy) AND

(cerebral hyperperfusion OR hyperperfusion syndrome OR postoperative hyperperfusion OR cerebral reperfusion injury) AND (hypertension OR blood pressure OR labile blood pressure OR BP control) AND (cerebral hypoperfusion OR impaired vasoreactivity OR cerebral autoregulation)

2. Supplemental Tables

2.1. **Supplemental Table S1.** PRISMA checklist for the meta-analysis of seizures following carotid endarterectomy.

Section and Topic	Item #	Checklist item	Location where item is reported
**TITLE**			
Title	1	Identify the report as a systematic review.	1
**ABSTRACT**			
Abstract	2	See the PRISMA 2020 for Abstracts checklist.	2
**INTRODUCTION**			
Rationale	3	Describe the rationale for the review in the context of existing knowledge.	3
Objectives	4	Provide an explicit statement of the objective(s) or question(s) the review addresses.	3
**METHODS**			
Eligibility criteria	5	Specify the inclusion and exclusion criteria for the review and how studies were grouped for the syntheses.	4-5
Information sources	6	Specify all databases, registers, websites, organisations, reference lists and other sources searched or consulted to identify studies. Specify the date when each source was last searched or consulted.	4-6
Search strategy	7	Present the full search strategies for all databases, registers and websites, including any filters and limits used.	4-5, Supplemental information
Selection process	8	Specify the methods used to decide whether a study met the inclusion criteria of the review, including how many reviewers screened each record and each report retrieved, whether they worked independently, and if applicable, details of automation tools used in the process.	4-5, PRISMA diagram (Figure 1)
Data collection process	9	Specify the methods used to collect data from reports, including how many reviewers collected data from each report, whether they worked independently, any processes for obtaining or confirming data from study investigators, and if applicable, details of automation tools used in the process.	4-5

Section and Topic	Item #	Checklist item	Location where item is reported
Data items	10a	List and define all outcomes for which data were sought. Specify whether all results that were compatible with each outcome domain in each study were sought (e.g. for all measures, time points, analyses), and if not, the methods used to decide which results to collect.	4-5
	10b	List and define all other variables for which data were sought (e.g. participant and intervention characteristics, funding sources). Describe any assumptions made about any missing or unclear information.	4-5
Study risk of bias assessment	11	Specify the methods used to assess risk of bias in the included studies, including details of the tool(s) used, how many reviewers assessed each study and whether they worked independently, and if applicable, details of automation tools used in the process.	4-5
Effect measures	12	Specify for each outcome the effect measure(s) (e.g. risk ratio, mean difference) used in the synthesis or presentation of results.	4-5
Synthesis methods	13a	Describe the processes used to decide which studies were eligible for each synthesis (e.g. tabulating the study intervention characteristics and comparing against the planned groups for each synthesis (item #5)).	4-5
	13b	Describe any methods required to prepare the data for presentation or synthesis, such as handling of missing summary statistics, or data conversions.	4-5
	13c	Describe any methods used to tabulate or visually display results of individual studies and syntheses.	4-5
	13d	Describe any methods used to synthesize results and provide a rationale for the choice(s). If meta-analysis was performed, describe the model(s), method(s) to identify the presence and extent of statistical heterogeneity, and software package(s) used.	4-5
	13e	Describe any methods used to explore possible causes of heterogeneity among study results (e.g. subgroup analysis, meta-regression).	4-5
	13f	Describe any sensitivity analyses conducted to assess robustness of the synthesized results.	4-5
Reporting bias assessment	14	Describe any methods used to assess risk of bias due to missing results in a synthesis (arising from reporting biases).	4-5
Certainty assessment	15	Describe any methods used to assess certainty (or confidence) in the body of evidence for an outcome.	4-5
**RESULTS**			
Study selection	16a	Describe the results of the search and selection process, from the number of records identified in the search to the number of studies included in the review, ideally using a flow diagram.	6
	16b	Cite studies that might appear to meet the inclusion criteria, but which were excluded, and explain why they were excluded.	6

Section and Topic	Item #	Checklist item	Location where item is reported
Study characteristics	17	Cite each included study and present its characteristics.	6
Risk of bias in studies	18	Present assessments of risk of bias for each included study.	6
Results of individual studies	19	For all outcomes, present, for each study: (a) summary statistics for each group (where appropriate) and (b) an effect estimate and its precision (e.g. confidence/credible interval), ideally using structured tables or plots.	6
Results of syntheses	20a	For each synthesis, briefly summarise the characteristics and risk of bias among contributing studies.	6
	20b	Present results of all statistical syntheses conducted. If meta-analysis was done, present for each the summary estimate and its precision (e.g. confidence/credible interval) and measures of statistical heterogeneity. If comparing groups, describe the direction of the effect.	6
	20c	Present results of all investigations of possible causes of heterogeneity among study results.	6
	20d	Present results of all sensitivity analyses conducted to assess the robustness of the synthesized results.	6
Reporting biases	21	Present assessments of risk of bias due to missing results (arising from reporting biases) for each synthesis assessed.	6
Certainty of evidence	22	Present assessments of certainty (or confidence) in the body of evidence for each outcome assessed.	6
**DISCUSSION**			
Discussion	23a	Provide a general interpretation of the results in the context of other evidence.	6-10
	23b	Discuss any limitations of the evidence included in the review.	6-10
	23c	Discuss any limitations of the review processes used.	6-10
	23d	Discuss implications of the results for practice, policy, and future research.	6-10
**OTHER INFORMATION**			
Registration and protocol	24a	Provide registration information for the review, including register name and registration number, or state that the review was not registered.	NA
	24b	Indicate where the review protocol can be accessed, or state that a protocol was not prepared.	NA
	24c	Describe and explain any amendments to information provided at registration or in the protocol.	NA

Section and Topic	Item #	Checklist item	Location where item is reported
Support	25	Describe sources of financial or non-financial support for the review, and the role of the funders or sponsors in the review.	NA
Competing interests	26	Declare any competing interests of review authors.	NA
Availability of data, code and other materials	27	Report which of the following are publicly available and where they can be found: template data collection forms; data extracted from included studies; data used for all analyses; analytic code; any other materials used in the review.	NA

2.2. **Supplemental Table S2.** MOOSE checklist for meta-analyses of observational studies included in the study on seizures post-carotid endarterectomy

Item No	Recommendation	Reported on Page No
Reporting of background should include		
1	Problem definition	3
2	Hypothesis statement	3
3	Description of study outcome(s)	3
4	Type of exposure or intervention used	3
5	Type of study designs used	3
6	Study population	3
Reporting of search strategy should include		
7	Qualifications of searchers (eg, librarians and investigators)	4
8	Search strategy, including time period included in the synthesis and key words	4
9	Effort to include all available studies, including contact with authors	4
10	Databases and registries searched	4
11	Search software used, name and version, including special features used (eg, explosion)	4
12	Use of hand searching (eg, reference lists of obtained articles)	4
13	List of citations located and those excluded, including justification	4
14	Method of addressing articles published in languages other than English	4
15	Method of handling abstracts and unpublished studies	4
16	Description of any contact with authors	4
Reporting of methods should include		
17	Description of relevance or appropriateness of studies assembled for assessing the hypothesis to be tested	4-6

18	Rationale for the selection and coding of data (eg, sound clinical principles or convenience)	4-6
19	Documentation of how data were classified and coded (eg, multiple raters, blinding and interrater reliability)	4-6
20	Assessment of confounding (eg, comparability of cases and controls in studies where appropriate)	4-6
21	Assessment of study quality, including blinding of quality assessors, stratification or regression on possible predictors of study results	4-6
22	Assessment of heterogeneity	4-6
23	Description of statistical methods (eg, complete description of fixed or random effects models, justification of whether the chosen models account for predictors of study results, dose-response models, or cumulative meta-analysis) in sufficient detail to be replicated	4-6
24	Provision of appropriate tables and graphics	Table 1, Figure 1-2
Reporting of results should include		
25	Graphic summarizing individual study estimates and overall estimate	Figure 2
26	Table giving descriptive information for each study included	Table 1
27	Results of sensitivity testing (eg, subgroup analysis)	-
28	Indication of statistical uncertainty of findings	9

2.3. **Supplemental Table S3.** Jaded analysis for methodological quality, risk of bias, and test for funding bias in the meta-analysis.

Jaded Analysis	Andereggen *et al.*	Buczek *et al.*	Wang *et al.*	Kieburtz *et al.*	Naylor *et al.*	Nielson *et al.*	Reigal *at al.*
Was the study randomised	0	1	1	0	0	0	0
Was the method of randomisation appropriate (not specified = 0)	0	1	1	0	0	0	0
Was the study described as being blinded?	0	0	0	0	0	0	0
Was the method of blinding appropriate	1	0	0	0	0	0	0
Was there a description of withdrawals and dropouts?	0	0	0	0	0	0	1
Was there a clear description of the inclusion/exclusion criteria	1	0	0	0	0	0	0
Was the method used to assess adverse events described?	1	1	1	1	1	1	1
Was the method of statistical analysis described?	1	1	1	0	0	0	0
**TOTALS (MJA_ROB)**	**4**	**4**	**4**	**1**	**1**	**1**	**2**
Funding Bias	0	2	0	0	0	0	0

Jaded Analysis	Bouri *et al.*	Wagner *et al.*	Dimakakos *et al.*	Karapanayiotides *et al.*	Jorgenson *et al.*	Abou-Chebl *et al.*	Sbarigia *at al.*
Was the study randomised	0	0	0	0	0	0	0
Was the method of randomisation appropriate (not specified = 0)	0	0	0	0	0	0	0

Was the study described as being blinded?	0	0	0	0	0	0	0
Was the method of blinding appropriate	0	0	0	0	0	0	0
Was there a description of withdrawals and dropouts?	0	0	0	0	0	0	0
Was there a clear description of the inclusion/exclusion criteria	1	0	1	1	1	1	1
Was the method used to assess adverse events described?	1	1	1	1	1	1	1
Was the method of statistical analysis described?	1	0	0	0	1	1	1
**TOTALS (MJA_ROB)**	**3**	**1**	**2**	**2**	**3**	**3**	**3**
Funding Bias	0	0	0	0	0	0	0

Jaded Analysis	Ogasawara *et al.*	Ascher *et al.*	Rockman *et al.*	Dalman *et al.*	Pennekamp *et al.*	Hirooka *et al.*
Was the study randomised	0	0	0	0	0	0
Was the method of randomisation appropriate (not specified = 0)	0	0	0	0	0	0

Was the study described as being blinded?	0	0	0	0	0	1
Was the method of blinding appropriate	0	0	0	0	0	1
Was there a description of withdrawals and dropouts?	1	1	1	1	1	0
Was there a clear description of the inclusion/exclusion criteria	1	1	1	1	1	1
Was the method used to assess adverse events described?	1	1	1	1	1	1
Was the method of statistical analysis described?	1	1	1	1	1	1
**TOTALS (MJA_ROB)**	**4**	**4**	**4**	**4**	**4**	**5**
Funding Bias	0	0	0	0	0	0

3. Supplemental Figures

3.1. **Supplemental Figure S1.** Influence of a single study on the meta-analysis of the association between pre-operative hypertension and seizures following carotid endarterectomy.

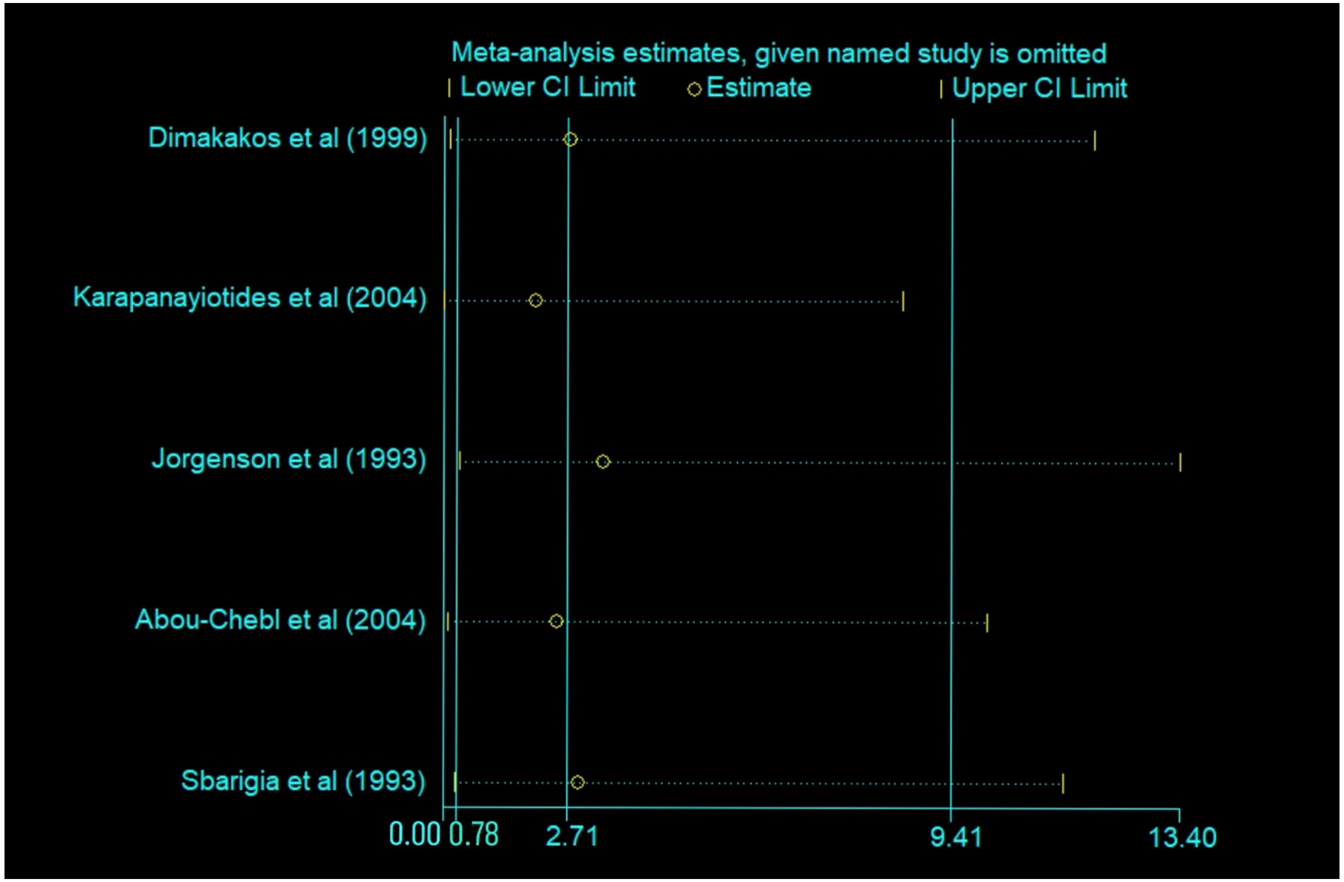

Abbreviations: CI: confidence interval

3.2. Supplemental Figure S2. Funnel plot with pseudo 95% confidence limits for the meta-analysis of seizures post-carotid endarterectomy.

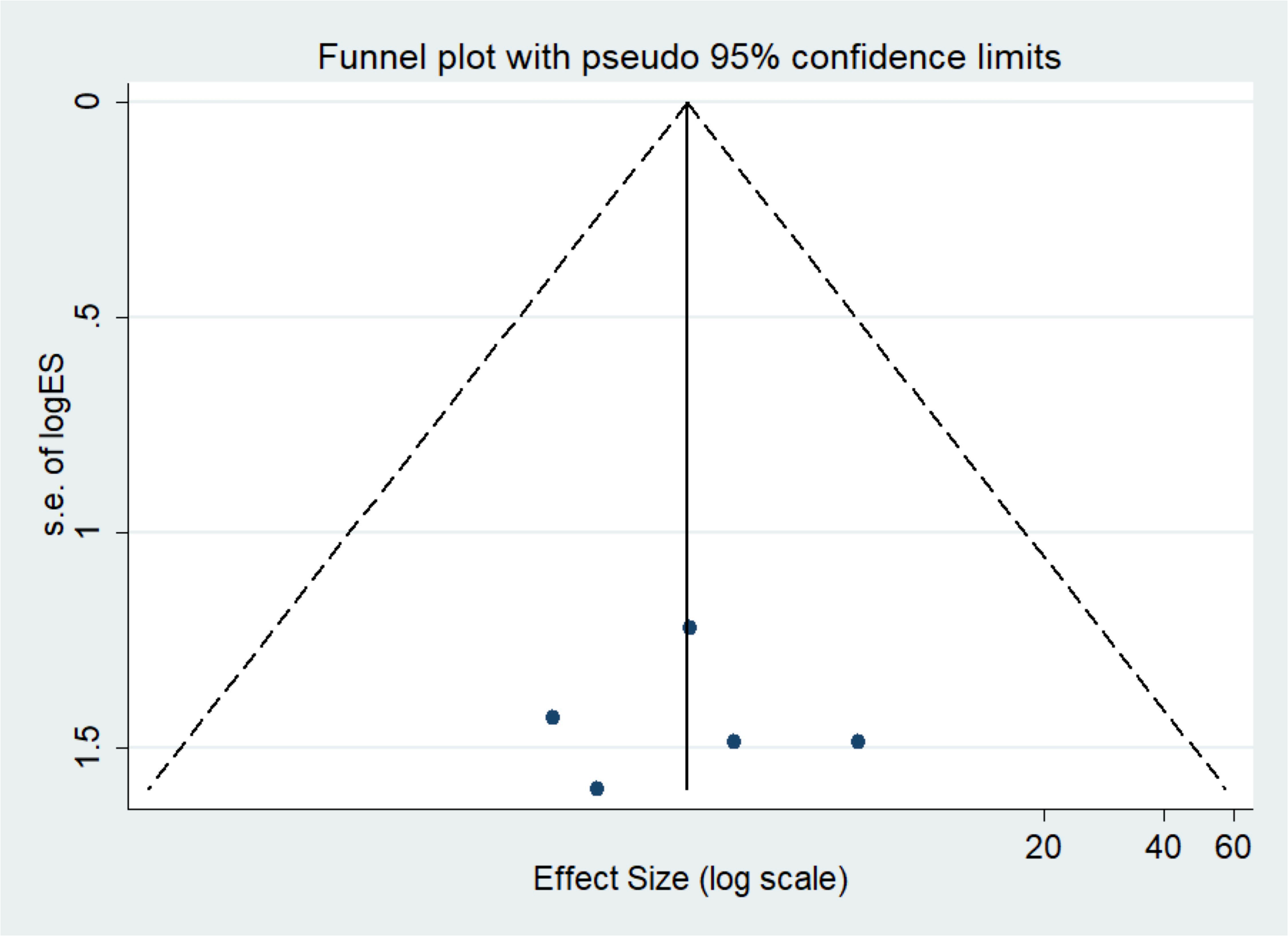

3.3. Supplemental Figure S3. Meta-analysis of pooled prevalence of seizures following carotid endarterectomy stratified by study design (prospective vs. retrospective).

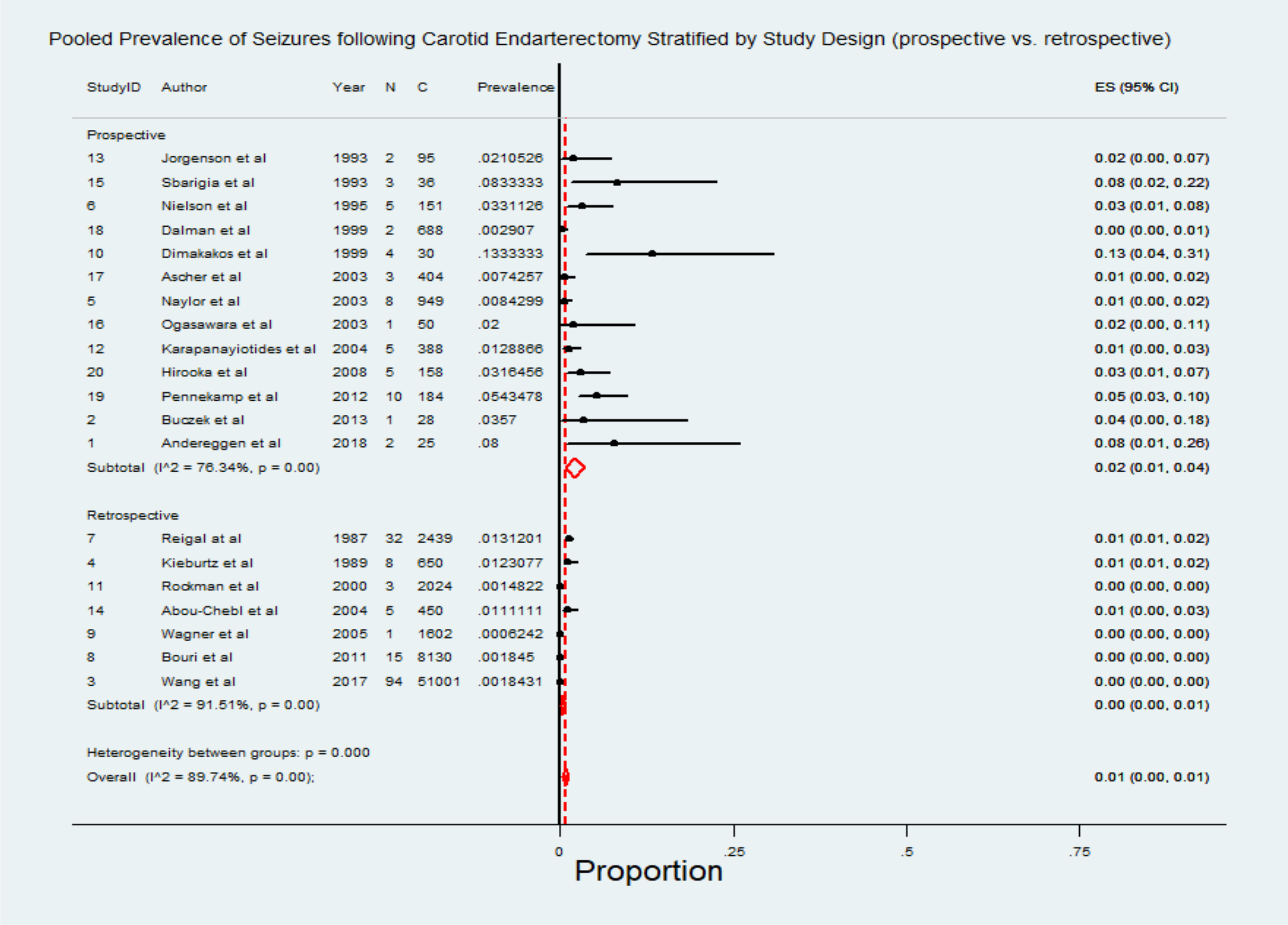

3.4. Supplemental Figure S4. Meta-analysis of pooled prevalence of pre-operative hypertension among patients who experienced seizures following carotid endarterectomy stratified by study design (prospective vs. retrospective).

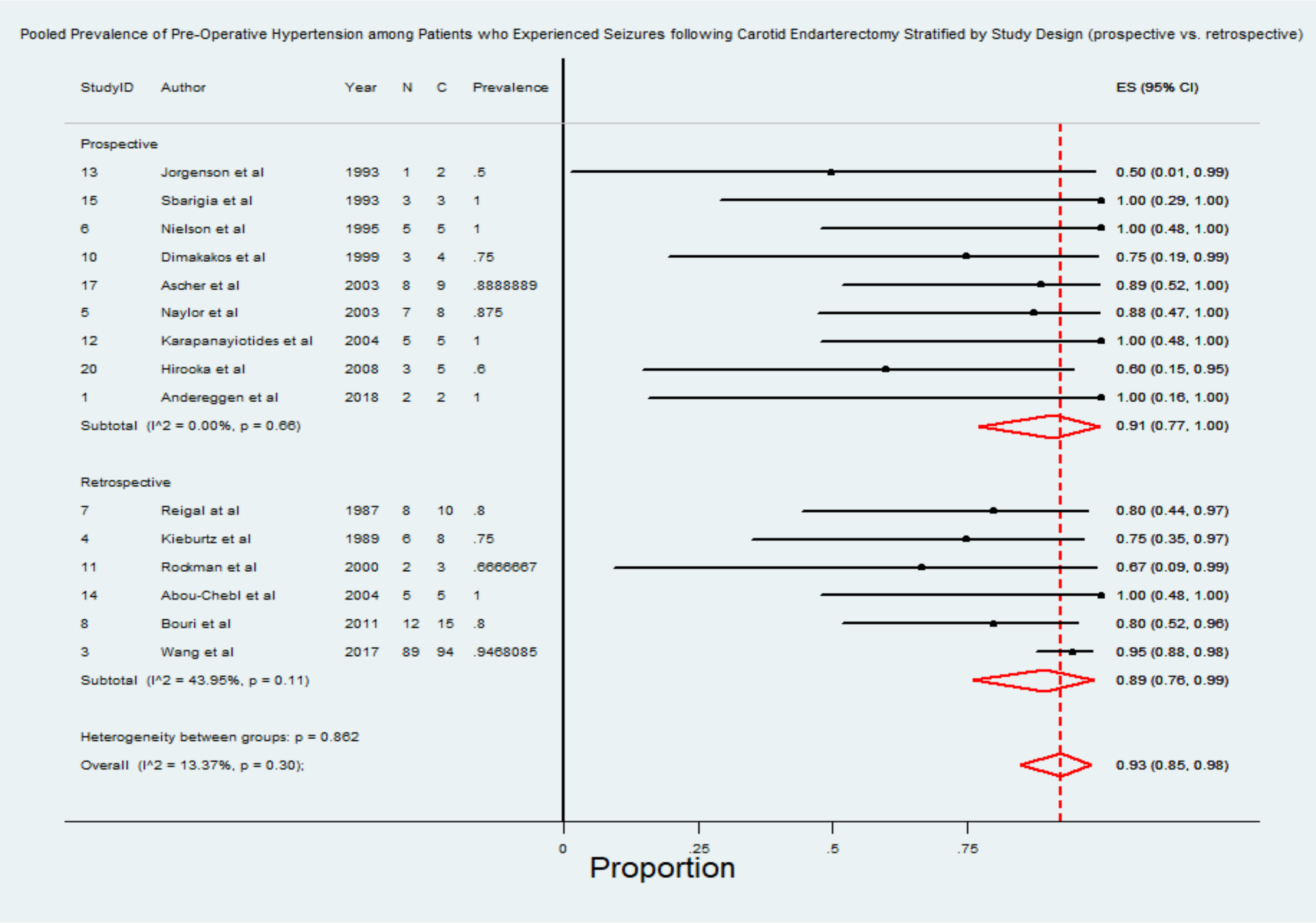

3.5. Supplemental Figure S5. Meta-analysis of pooled prevalence of cerebral hyperperfusion syndrome following carotid endarterectomy stratified by study design (prospective vs. retrospective).

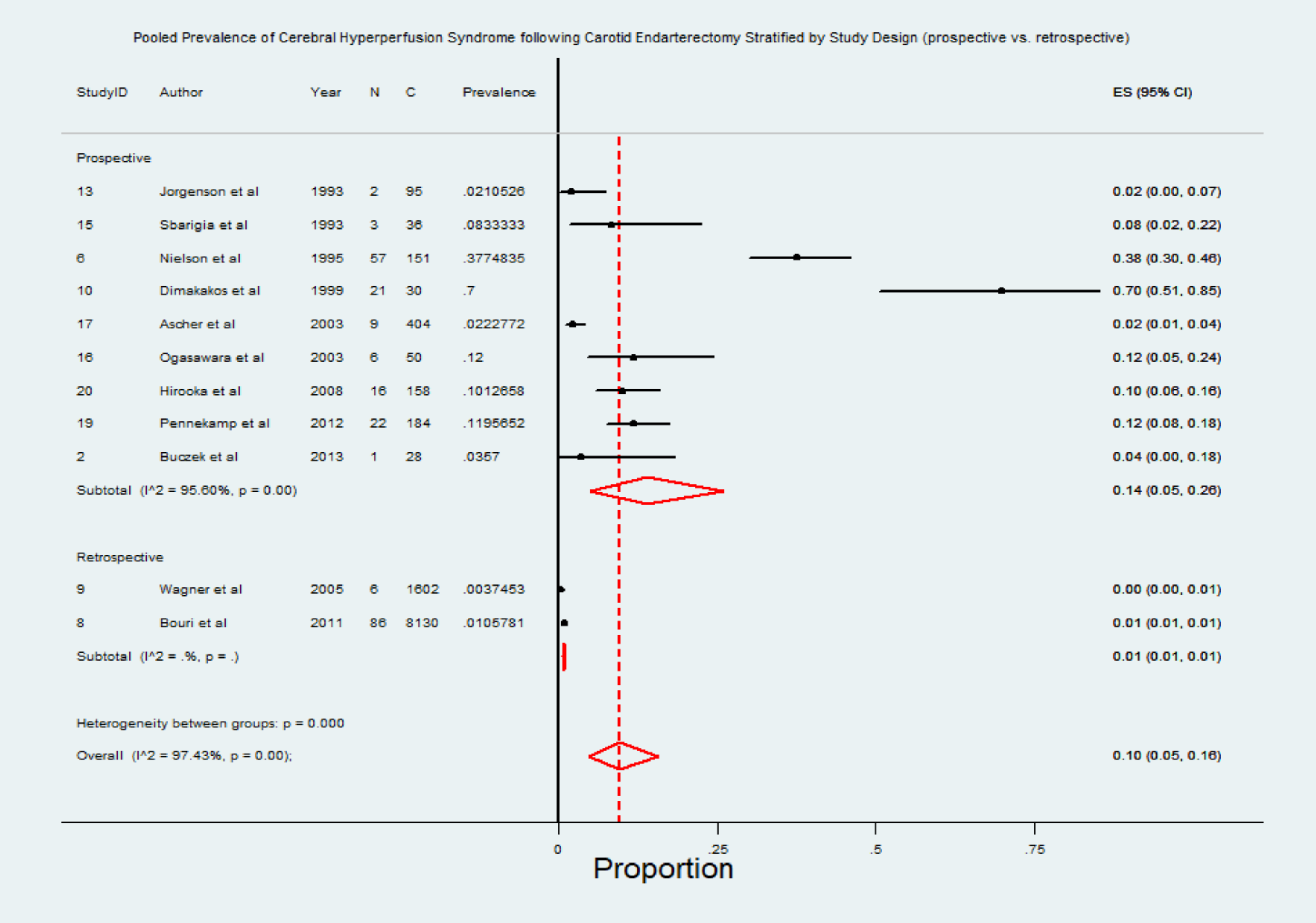

Supplement: Supplementary file 1 [file diagnostics-15-00006-s001.zip › Supplemental Information SI1.pdf]
