# Supplementary material for: Seizures Following Carotid Endarterectomy: A Comprehensive Meta-Analysis of 69,479 Patients and Evidence-Based Recommendations for Perioperative Care
Source: Diagnostics (Basel). 2024 Dec 24;15(1):6. doi: 10.3390/diagnostics15010006 (PMC11840276; doi:10.3390/diagnostics15010006)
Supplement: Supplementary file 2 [file diagnostics-15-00006-s002.zip › Supplemental Information SI2_SMART-CEA Checklist.pdf]

SMART-CEA Checklist: A Practical Guide for Screening and Management of Seizures and Associated Complications Post-Carotid Endarterectomy

SMART-CEA Checklist for Screening and Management of Seizures and Associated Complications Post-Carotid Endarterectomy			
Step	**Action**	**Description**	**Tick/Check**
**Preoperative Screening**			
1. Assess patient history	Evaluate for prior seizures, epilepsy, stroke, transient ischemic attack (TIA), or uncontrolled hypertension.	Identify high-risk patients based on medical history.	☐
2. Optimize blood pressure (BP)	Ensure preoperative BP control, targeting <130/80 mmHg.	Optimize BP to reduce the risk of cerebral hyperperfusion syndrome and seizures.	☐
3. Smoking cessation counseling	Advise and support smoking cessation prior to surgery.	Implement smoking cessation programs to reduce vascular risk and postoperative complications.	☐
4. Evaluate comorbidities	Screen for hypertension, coronary artery disease (CAD), diabetes, and hyperlipidemia.	Identify and manage comorbidities that may increase perioperative risk.	☐
5. Preoperative imaging	Perform transcranial Doppler (TCD) or other imaging to assess cerebral autoregulation and perfusion status.	Use advanced imaging (e.g., TCD, MRI, CT perfusion) to assess cerebral vasoreactivity and identify high-risk patients.	☐
**Intraoperative Management**			
6. Maintain hemodynamic stability	Use anesthetic protocols to maintain normotension and normocarbia during surgery.	Tailor anesthesia protocols to individual patient risk profiles to reduce hemodynamic fluctuations and seizure risk.	☐
7. Monitor cerebral perfusion	Use TCD monitoring intraoperatively to detect hyperperfusion or impaired autoregulation.	Routine use of TCD monitoring during surgery to identify patients at risk of cerebral hyperperfusion syndrome.	☐
Postoperative Monitoring			
8. Monitor for seizures	Observe for seizures or neurological changes during the first 1–8 days postoperatively.	Closely monitor high-risk patients, particularly those with a history of stroke or TIA.	☐
9. Monitor BP closely	Maintain BP within target range (<140/90 mmHg) using antihypertensive agents as needed.	Gradual BP reduction is recommended in patients with severe symptomatic carotid stenosis to minimize complications.	☐
10. Assess for hyperperfusion syndrome	Monitor for symptoms such as unilateral headache, confusion, focal neurological deficits, or seizures.	Early identification of cerebral hyperperfusion syndrome (CHS) is critical to prevent seizures and other complications.	☐
**Management of Seizures and Complications**			
11. Treat seizures promptly	Administer benzodiazepines (e.g., diazepam) for acute seizures.	Prompt treatment of seizures is essential to prevent further complications.	☐
12. Manage cerebral hyperperfusion syndrome	Use antihypertensive agents (e.g., labetalol) and corticosteroids (e.g., dexamethasone) as needed.	Treat CHS promptly to manage symptoms and prevent seizures.	☐
13. Perform imaging if seizures occur	Conduct CT or MRI to rule out intracranial hemorrhage or other complications.	Imaging is critical to identify underlying causes of seizures, such as hemorrhage or ischemia.	☐
14. Consider anticonvulsants in high-risk patients	Prophylactic use of anticonvulsants in patients with prior seizures, CHS, or severe hypertension.	Conditional recommendation based on limited evidence; consider for high-risk patients.	☐
**Long-Term Follow-Up**			
15. Standardize follow-up	Schedule regular follow-ups to monitor for delayed seizures or neurological complications.	Long-term follow-up is essential to capture delayed events and assess progression to epilepsy or cognitive decline.	☐
16. Educate patients	Provide education on recognizing seizure symptoms and when to seek medical attention.	Educate patients and caregivers on early symptoms of CHS and seizures to ensure timely intervention.	☐
17. Consider advanced monitoring	Use advanced imaging (e.g., quantitative MRI) or biomarkers for high-risk patients.	Emerging evidence supports the use of advanced imaging and biomarkers for risk stratification and monitoring.	☐
18. Monitor for cognitive decline	Implement long-term monitoring for cognitive decline and neurological complications, particularly in patients with postoperative seizures.	Conditional recommendation based on limited evidence linking seizures and CHS to cognitive decline.	☐
**Multidisciplinary Approach**			
19. Adopt a multidisciplinary approach	Involve neurologists, vascular surgeons, anesthesiologists, and radiologists to optimize perioperative care.	Collaboration among specialists ensures comprehensive risk assessment, monitoring, and management of complications.	☐

SMART-CEA stands for: **S – Screening:** Assess risk factors such as hypertension, prior seizures, TIA, and smoking; **M – Monitoring:** Monitor cerebral perfusion and blood pressure intraoperatively and postoperatively; **A – Assessing:** Assess for hyperperfusion syndrome and seizure symptoms; **R – Responding:** Respond promptly to seizures and complications with appropriate interventions; **T – Tailoring:** Tailor long-term follow-up and patient education for seizure prevention and management; **CEA** – Carotid Endarterectomy. The SMART-CEA checklist provides a practical, step-by-step guide for clinicians to ensure comprehensive screening, monitoring, and management of seizures and associated complications in patients undergoing CEA. Each step includes a tick/check option for easy tracking and implementation in clinical workflows.

Abbreviations: AF – atrial fibrillation; **HL** – hyperlipidaemia; **HTN** – hypertension; **CAD** – coronary artery disease; **TIA** – transient ischaemic attack; **CEA** – carotid endarterectomy; **TCD** – transcranial Doppler; **BP** – blood pressure; **CT** – computed tomography; **MRI** – magnetic resonance imaging.

Version 1.1 [Dated December 23, 2024]

Developed by Sonu M. M. Bhaskar, MD PhD FANA
